# Ultra‐Micro Powder From Edible Plants (*Pueraria lobata*, *Cornus officinalis*, *Cistanche deserticola*, and *Dendrobium officinale*) Reshapes Intestinal Flora and Attenuates Liver Injury in Alcohol‐Induced Mice

**DOI:** 10.1002/fsn3.71890

**Published:** 2026-05-13

**Authors:** Jia‐Yu Huang, Wei‐Feng Huang, Xin‐Yu Wang, Jie Cheng, Guang‐Hui Xu, Li‐Tao Yi

**Affiliations:** ^1^ Department of Chemical and Pharmaceutical Engineering, College of Chemical Engineering Huaqiao University Xiamen Fujian China; ^2^ Department of Gastroenterology and Hepatology The First Affiliated Hospital of Xiamen University, School of Medicine, Xiamen University Xiamen Fujian China; ^3^ Xiamen Medicine Research Institute Xiamen Fujian China; ^4^ Institute of Pharmaceutical Engineering Huaqiao University Xiamen Fujian China; ^5^ Fujian Provincial Key Laboratory of Biochemical Technology Huaqiao University Xiamen Fujian China

**Keywords:** *Cistanche deserticola*, *Cornus officinalis*, *Dendrobium officinale*, intestinal flora, liver injury, *Pueraria lobata*

## Abstract

Alcohol consumption is a critical global health concern linked to liver injury. This study evaluated the hepatoprotective effects of an edible ultra‐micro powder—formulated from *Pueraria lobata* , *Cornus officinalis* , *Cistanche deserticola* , and *Dendrobium officinale*—against alcohol‐induced damage in mice. Mice were randomly assigned to seven groups: Normal, Alcohol, Silymarin, KingDrink, and ultra‐micro powder (low, medium, and high doses) for 1 week of daily treatment. The medium dose (1 g/kg) was most effective, reducing serum aspartate aminotransferase and triglyceride levels, lowering hepatic malondialdehyde, and maintaining liver architecture with minimal microvesicular steatosis. The powder increased alcohol dehydrogenase, acetaldehyde dehydrogenase, and superoxide dismutase activities, thereby enhancing alcohol metabolism and antioxidant defense. Additionally, it suppressed TLR4/NF‐κB and NLRP3/ASC signaling, indicating an anti‐inflammatory mechanism. 16S rRNA sequencing revealed gut microbiota modulation, specifically reducing Proteobacteria and Escherichia‐Shigella while enriching Lactobacillus. Correlation analysis linked Verrucomicrobiota and Lactobacillus to improved liver function. Collectively, this powder exerts hepatoprotection through antioxidant, anti‐inflammatory, and microbiota‐modulating actions, highlighting its potential as a functional food for preventing alcoholic liver injury.

## Graphical abstract

Ultra‐micro powder from edible plants ( *Pueraria lobata* , *Cornus officinalis* , *Cistanche deserticola* , and *Dendrobium officinale*) attenuates alcoholic liver injury by regulating the gut‐liver axis. It primarily reshapes the intestinal microbiota by restoring beneficial *Lactobacillus* populations and suppressing pathogenic bacteria like Proteobacteria and *Escherichia‐Shigella*. Consequently, in the liver, the powder mitigates injury by enhancing alcohol metabolism through ALDH upregulation, boosting antioxidant defenses, and inhibiting TLR4/NLRP3‐mediated pro‐inflammatory cascades to preserve overall hepatic architecture.

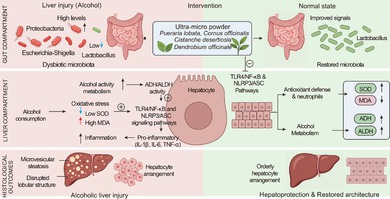

## 1 Introduction

Alcohol abuse and its systemic consequences impose a substantial burden on global public health systems. Chronic consumption induces a range of disorders, notably alcoholic liver injury, which can progress from simple steatosis to severe conditions like hepatitis, fibrosis, and cirrhosis (Hosseini et al. 2019). Consequently, there is an urgent need for effective strategies to prevent and treat alcohol‐related liver damage.

Alcoholic liver injury arises from the complex interplay between ethanol metabolism, oxidative stress, inflammation, and lipid metabolic dysfunction (Hyun et al. 2021). Chronic consumption generates reactive oxygen species (ROS), inducing oxidative stress that triggers inflammatory cascades and hepatocellular damage. Furthermore, alcohol‐induced metabolic dysregulation promotes lipid accumulation and hepatosteatosis, further compromising liver function (Jeon and Carr 2020). Current therapeutic strategies are limited, primarily relying on abstinence (German et al. 2021). Given high relapse rates, effective pharmacological interventions are urgently needed to ameliorate damage from excessive alcohol consumption.

In recent years, there has been growing interest in exploring natural compounds with potential hepatoprotective effects (Madrigal‐Santillan et al. 2014). Among these, edible plants have emerged as promising sources of bioactive compounds with diverse health benefits. *Pueraria lobata* , commonly known as kudzu root, has been traditionally used in Eastern medicine for its various health benefits. It exhibits antioxidant, anti‐inflammatory, and hepatoprotective properties, attributed to its rich content of isoflavones, particularly puerarin (Zhao et al. 2022). Puerarin could attenuate alcohol‐induced oxidative stress, inflammation, and liver injury in preclinical models (Keskin Alkac et al. 2022). *Cornus officinalis* possesses potent antioxidant and anti‐inflammatory properties (Lee et al. 2012). Its bioactive compounds, including iridoids, flavonoids, and triterpenes, have shown hepatoprotective effects against various liver diseases, including alcoholic liver injury, which could be associated with the regulation of oxidative stress, inflammation, and lipid metabolism (An et al. 2022). *Cistanche deserticola* , a desert‐dwelling parasitic plant, has gained attention for its pharmacological activities, including antioxidant, anti‐inflammatory, and anti‐apoptotic effects. Its active constituents, such as glycosides and polysaccharides, contribute to its hepatoprotective properties (Wang, Wang, Sun, et al. 2025; Yuan et al. 2026). A previous study has reported the potential of *Cistanche deserticola* in attenuating alcohol‐induced liver damage by reducing oxidative stress and inflammatory responses (Yan et al. 2023). *Dendrobium officinale*, an orchid species, contains polysaccharides, alkaloids, and phenolic compounds that possess antioxidant and anti‐inflammatory properties. *Dendrobium officinale* has shown hepatoprotective effects by reducing lipid peroxidation, inflammation, and apoptosis in liver tissues (Hui et al. 2023; Jing et al. 2022). However, it is unclear how inflammatory signaling pathways and intestinal flora are associated with the protective role in alcohol‐induced liver injury except for three publications showing the improvement of *Pueraria lobata* and *Dendrobium officinale* on TLR4 signaling or intestinal flora in hepatoprotective activity (Feng et al. 2019; K. Yang et al. 2020; Zhao et al. 2022). These four plants have been approved to list in the Directory of Medicinal and Edible Homologous Plants by the National Medical Products Administration of China. According to Traditional Chinese Medicine (TCM) theory, *Pueraria lobata* , *Cornus officinalis* , *Cistanche deserticola* , and *Dendrobium officinale* nutritively target the stomach, liver, colon, and kidney, respectively. Consequently, a formulation of these four plants may provide systemic health benefits through multi‐organ regulation.

Based on the promising hepatoprotective properties of the individual components, this study aims to comprehensively evaluate the potential of a unique edible ultra‐micro powder containing *Pueraria lobata, Cornus officinalis, Cistanche deserticola, and Dendrobium officinale* in mitigating alcoholic liver injury. The study investigates the effects of the ultra‐micro powder on alcohol tolerance, sleep and sobriety time, as well as biochemical and molecular markers of liver damage.

## 2 Materials and Methods

### 2.1 Animals

SPF grade Kunming male mice (body mass 24–28 g) were purchased from Shanghai Slac Laboratory Animal Centre (Shanghai, China). Animals were housed in the standard animal facility at Huaqiao University with four mice per cage under controlled environmental conditions: temperature (22°C ± 2°C), humidity (50% ± 5%), and a 12 h light/dark cycle. All mice had ad libitum access to standard laboratory chow and water. Following a one‐week acclimatization period, mice were randomly assigned to experimental groups using a random number generator. All procedures were approved by the Institutional Animal Care and Use Committee of Huaqiao University (Approval No. A2023027) and conducted in strict accordance with the guidelines set by the China Council on Animal Care. During the experiment, mice were randomly divided into groups using a random number table. To ensure objective assessment, histopathological and biochemical analyses were performed in a blinded manner.

### 2.2 Preparation of Plant Ultra‐Micro Powder

*Pueraria lobata* (Collected from Hengfeng, Jiangxi Province), *Cornus officinalis* (Collected from Yangcheng, Shanxi Province), *Cistanche deserticola* (Collected from Alashan, Inner Mongolia Province), and *Dendrobium officinale* (Collected from Huoshan, Anhui Province) were purchased from Bozhou Kang Yi Drink Biotechnology Co. (Bozhou, China). The plants were identified by Cheng‐Fu Li in Xiamen Hospital of Traditional Chinese Medicine. 500 g of each of Pueraria lobata, Cornus officinalis, Cistanche deserticola, and Dendrobium officinale were processed into powder by ultra‐micro crusher, passed through a 0.180 mm (80 mesh) sieve, mixed well by blender, dried at 60°C for about 12 h until the moisture was less than 5%, sterilized at 80°C for 8 min, sealed and used in proportion, and prepared into a mixed solution of 0.1 g/mL when used.

### 2.3 Characterization of Ultra‐Micro Powder

To identify the major chemical constituents of the ultra‐micro powder, a qualitative analysis was performed. The analysis was performed on a Thermo Vanquish UPLC system (Thermo Fisher Scientific, USA) coupled to a Thermo Orbitrap Exploris 120 mass spectrometer (Thermo Fisher Scientific, USA) with an electrospray ionization (ESI) source operating in both positive and negative modes. Separation was achieved using an ACQUITY UPLC HSS T3 column (2.1 × 100 mm, 1.8 μm; Waters, USA) at 40°C, with a flow rate of 0.3 mL/min and an injection volume of 5 μL. For positive ion mode, the mobile phase consisted of 0.1% formic acid in acetonitrile (B2) and 0.1% formic acid in water (A2); for negative ion mode, acetonitrile (B3) and 5 mM ammonium formate in water (A3) were used. Both modes employed an identical gradient program: 0–1 min, 8% B; 1–8 min, 8%–98% B; 8–10 min, 98% B; 10–10.1 min, 98%–8% B; 10.1–12 min, 8% B. MS parameters included spray voltages of 3.50 kV (positive) and −2.50 kV (negative), sheath/auxiliary gas flows of 40/10 arb, and a capillary temperature of 325°C. Full‐scan MS data (resolution: 60,000; *m/z* 100–1000) were acquired, with the top four ions selected for HCD fragmentation (30% NCE; MS/MS resolution: 15,000) and dynamic exclusion to minimize redundancy.

Raw mass spectrometry data were converted to mzXML format using MSConvert (ProteoWizard, v3.0.8789). Peak detection, filtering, and alignment were conducted via the R XCMS package with the following parameters: bw = 2, ppm = 15, peakwidth = c(5, 30), mzwid = 0.015, mzdiff = 0.01, and method = “centWave”. Systematic errors were eliminated through total peak area normalization. Qualitative metabolite identification was performed by searching public databases including HMDB, LipidMaps, KEGG, and ChEBI, alongside a custom‐built standard library. Primary identification was achieved by determining molecular weights from parent ion mass‐to‐charge ratios (m/z), predicting molecular formulas via mass deviation (ppm) and adduct information, and database matching. Concurrently, secondary metabolites were identified by matching detected secondary spectra with fragment ion data in the databases.

### 2.4 Reagents

The kits of glutamic oxaloacetic transaminase (AST, BC1565), glutamic alanine transaminase (ALT, BC1555), superoxide dismutase (SOD, BC0175), ethanol dehydrogenase (ADH, BC1085), acetaldehyde dehydrogenase (ALDH, BC0755), glutathione (GSH, BC1175) and malondialdehyde (MDA, BC0025) were purchased from Beijing Solarbio Technology Co. (Beijing, China). TLR4 Antibody (BP64876), pNF‐κB Antibody (BP65429), NLRP3 Antibody (BP63174), COX‐2 Antibody (BP61049), GAPDH Antibody (BP69330), interleukin‐1 beta (IL‐1β) ELISA kit (BP80277), interleukin‐6 (IL‐6) ELISA kit (BP80293), and tumor necrosis factor‐alpha (TNF‐α) ELISA kit (BP80317) were purchased from Purdue Bioscience Inc. (New York, USA). ASC Antibody (sc‐514,414) was purchased from Santa Cruz Inc. (Santa Cruz, USA). Donkey Anti‐Rabbit IgG H&L (Alexa Fluor 488) preadsorbed Antibody (ab150061) and Donkey Anti‐Mouse IgG H&L (Alexa Fluor 647) preadsorbed Antibody (ab150111) were purchased from Abcam Inc. (Cambridge, USA).

### 2.5 Alcohol Tolerance Test in Mice

Mice were randomly divided into four groups of eight mice each. The mice were gavaged with 56° white wine (Hongxing Erguotou) at doses of 0.10, 0.12, 0.14, and 0.16 mL/10 g after 12 h of fasting without water for 1 week of acclimatization feeding. The dose corresponding to the maximum intoxication rate and the minimum mortality rate of the mice was determined to be the best tolerated alcohol gavage amount. The maximum intoxication rate and the minimum mortality rate of the mice were determined to be the best tolerated dose. To better simulate the physiological stress of commercial spirits consumption, mice were gavaged with commercial white wine instead of ethanol. This model was selected to reflect a realistic alcoholic beverage consumption scenario rather than a pure ethanol exposure model.

### 2.6 Behavioral Experiments

Before gavage, each mouse was weighed and numbered, and the dose of drug and alcohol was calculated based on body weight. The following behavioral endpoints were recorded: (1) Alcohol tolerance time: The interval between the administration of alcohol and the loss of righting reflex. (2) Sleep time: Defined as the point at which the mouse was unable to right itself within 30 s when placed on its back. (3) Sobriety time: Defined as the interval from sleep until the mouse successfully regained its upright position.

### 2.7 Alcohol‐Induced Liver Injury in Mice

Mice were randomly divided into seven groups of eight mice each. They were divided into Normal group, alcohol group, silymarin group, KingDrink group and ultra‐micro powder at low, medium and high dose groups. The mice in the Normal group were gavaged with distilled water at a dose of 0.10 mL/10 g. The mice in the alcohol group were gavaged with the same volume of distilled water for 30 min and then given 56° white wine at a dose of 0.14 mL/10 g. Based on the alcohol content (56% v/v) and the density of ethanol (0.789 g/mL), this corresponds to an ethanol dose of approximately 6.19 g/kg. The low, medium and high dose groups of ultra‐micro powder were fed with 0.5, 1.0, and 2.0 g/kg of ultra‐micro powder, respectively. 30 min later, 56° white wine was given at a dose of 0.14 mL/10 g. Silymarin and KingDrink groups were given at a dose of 70 mg/kg of Silymarin or 0.6 g/kg KingDrink (including oyster extract and taurine), respectively, followed by 56° white wine given. Drugs and alcohol were administrated for 1 week. This modeling approach, utilizing a commercial spirit rather than pure ethanol, was selected to simulate realistic human consumption patterns and has been previously validated in models of alcoholic liver disease (Wang, Wang, Li, et al. 2025; Xu et al. 2020).

### 2.8 Sample Collection

1. Fasted without water for 16 h after the last gavage and weighed before execution. Orbital blood sampling was performed and approximately 800 μL of each blood sample was taken in a 1.5 mL EP tube, centrifuged at 10,000 g for 10 min at 4°C and 200 μL of supernatant was stored in a new EP tube. The serum obtained was separated and serum AST, ALT and TG levels were determined according to the kit instructions.
2. The livers were dissected, quickly removed, immediately rinsed with pre‐cooled saline, blotted dry with filter paper, and weighed to calculate the liver index. The formula for calculating liver index: Liver index/% = mouse liver mass/mouse body weight × 100%.
3. A portion of mouse liver tissue was cut and saline was used as a buffer to prepare a 10% liver tissue homogenate, after which the supernatant was centrifuged at 4°C for 10 min. SOD, ADH, ALDH, GSH, and MDA in liver tissues were determined according to the method in the kits.
4. A portion of mouse liver was washed with pre‐cooled PBS and added to a glass homogenizer with PBS on ice. The homogenate was centrifuged at 4°C for 10 min and the supernatant was taken for assay. After the protein concentration was determined by BCA method, the cytokine content was determined by ELISA kit according to the manufacturer's instructions.
5. A portion of mouse liver was taken, fixed in 4% paraformaldehyde fixative and embedded in paraffin, and made into liver histopathological sections, stained with HE, observed under a microscope at 400 times, and photographed for analysis. To objectively quantify liver injury, three non‐overlapping fields per section were randomly selected and photographed at 400 times magnification. Pathological scores were assigned by a pathologist blinded to the treatment groups based on the following criteria: (i) Steatosis (0: < 5%; 1: 5%–33%; 2: 34%–66%; 3: > 66% of the field); (ii) Inflammation (0: none; 1: 1–2 foci; 2: 3–4 foci; 3: > 4 foci); and (iii) Hepatocyte arrangement/necrosis (0: normal; 1: mild focal disruption; 2: moderate disruption; 3: severe architectural loss).
6. A portion of mouse liver was subjected to gradient dehydration treatment by soaking in 10%, 20%, and 30% sucrose solution for 24 h. The tissues were then embedded with OCT, snap frozen in liquid nitrogen, cut into 15 μm slices with a slicer, and stored in a −80°C refrigerator for subsequent immunofluorescence experiments.
7. Feces were collected from the end of the colon of mice and subsequently subjected to intestinal flora assay.

### 2.9 Liver Immunofluorescence

To expose antigens masked by fixation and enhance their immunoreactivity, antigen retrieval was performed for the tissue sections. Then, the non‐specific binding sites on the tissue sections are blocked using a blocking solution. The sections were then incubated with primary antibodies (all dilution at 1:100) specific to the target proteins overnight at 4°C. After washing off excess primary antibody, the tissue sections were incubated with fluorochrome‐conjugated secondary antibodies. These secondary antibodies recognize the primary antibody's host species and allow for visualization under fluorescence microscopy. Subsequently, nuclear counterstaining with a DNA‐binding dye, DAPI, was added for visualizing cell nuclei. Finally, mounting medium is applied to preserve the fluorescence and cover the tissue sections with a coverslip. The immunofluorescence‐labeled liver sections were visualized using a fluorescence microscope (Leica TCS SP8). Image acquisition was followed by data analysis, which included quantification of fluorescence intensity using image analysis software Imaris.

### 2.10 16S RNA Intestinal Flora Assay

Total DNA was extracted from the stool samples using the E.Z.N.A. Soil DNA Kit (Omega Bio‐tek, USA) according to the manufacturer's protocol. The V3‐V4 region of the 16S rRNA gene was amplified using universal primers. The PCR reaction mixture contained the DNA template, primers, dNTPs, buffer, and high‐fidelity DNA polymerase, with thermal cycling conditions optimized for target amplification. Amplicons were purified using a magnetic bead‐based method, and index/barcode sequences were added during a second round of PCR to enable multiplexing. The prepared libraries were quantified, pooled in equimolar concentrations, and subjected to high‐throughput sequencing on the Illumina PE300/PE250 platform (Illumina, USA). The sequencing generated 2 × 300 bp paired‐end reads. Raw data underwent rigorous quality control via the QIIME2 pipeline (version 2022.2) to remove low‐quality reads (*Q* < 20), adapter sequences, and PhiX contamination, targeting a depth of at least 30,000 clean reads per sample. Bioinformatics processing included merging paired‐end reads and quality filtering, followed by clustering into operational taxonomic units (OTUs) based on a 97% sequence similarity threshold. While Amplicon Sequence Variants (ASVs) offer higher resolution, the OTU‐based approach was utilized here to assess broad community shifts in diversity and richness. Representative sequences for each OTU were taxonomically assigned by comparison against the SILVA reference database (version 138). The resulting taxonomic profiles were used to evaluate microbial composition across the experimental groups.

### 2.11 Statistical Analyses

The data were expressed as mean ± standard error. A sample size of *n* = 8 per group was utilized based on established protocols for alcohol‐induced liver injury models to ensure sufficient statistical power for detecting differences in biochemical and molecular markers. Before statistical analysis, the normality of the data distribution was verified using the Shapiro–Wilk test, and the homogeneity of variances was assessed with the Brown‐Forsythe test. For data meeting these assumptions, differences between multiple groups were compared using one‐way ANOVA followed by Tukey's post hoc test for pairwise comparisons. For the quantitative histological scoring introduced in the previous revision, six representative samples per group were utilized to ensure sections were free of paraffin‐related technical artifacts and suitable for blinded assessment. Histological scores were analyzed by the Kruskal‐Wallis test. A *p* < 0.05 indicated a significant difference.

## 3 Results

### 3.1 The Constituent Profile of the Ultra‐Micro Powder by UPLC‐MS

**FIGURE 1:**
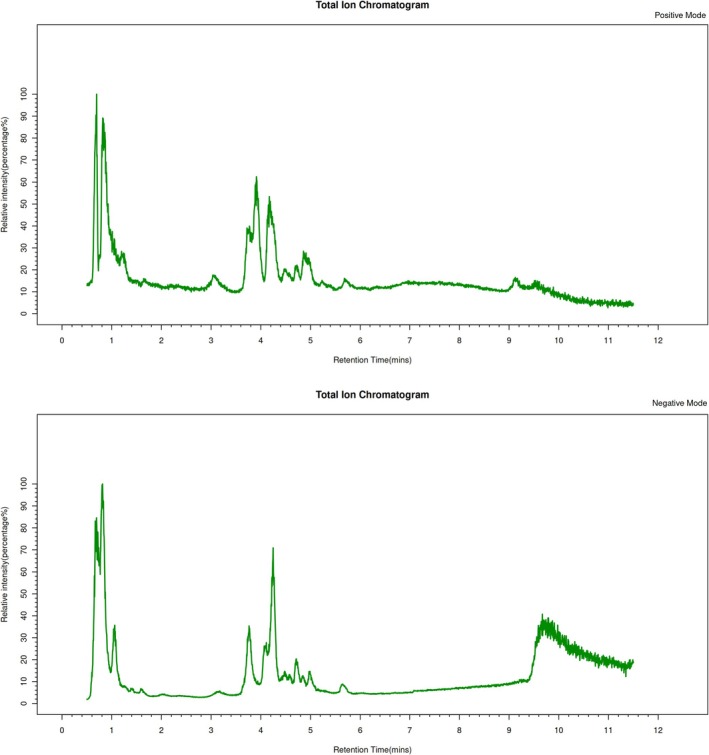
Total ion chromatogram of ultra‐micro powder by UPLC‐MS.

Qualitative profiling by UPLC‐MS revealed a total of 51 major constituents across the four plant components (File S1). Briefly, the chemical constituents of the ultra‐micro powder were characterized by UPLC‐MS. According to Figure 1 and Table 1, the results indicated that there were 15 constituents from *Pueraria lobata* , 13 constituents from *Cornus officinalis* , 18 constituents from *Cistanche deserticola* , and 5 constituents from *Dendrobium officinale*. Among the components, Puerarin was the characteristic constituent of *Pueraria lobata* . Loganin and Cornuside are the characteristic constituents of *Cornus officinalis* . Cistanoside f and Verbascoside are the characteristic constituents of *Cistanche deserticola* .

**TABLE 1:** The chemical constituents of the edible ultra‐micro powder by UPLC‐MS.

Chemical name	RT (s)	Exact_mass	Formula	CAS	Origin
Formononetin 7‐(6′‐malonylglucoside)	39.9	516.12677	C_25_H_24_O_12_	34,232‐16‐1	*Pueraria lobata*
Succinic acid	41.1	118.02661	C_4_H_6_O_4_	110‐15‐6	*Cistanche deserticola*
2′‐Hydroxydaidzein	44.6	270.052821	C_15_H_10_O_5_	7678‐85‐5	*Pueraria lobata*
Hyacinthin	46.9	595.1451592	C_30_H_27_O_13+_	56,767‐17‐0	*Cistanche deserticola*
Eugenol	64.9	164.0837252	C_10_H_12_O_2_	97‐53‐0	*Cistanche deserticola*
Protocatechuic acid	66.1	154.02661	C_7_H_6_O_4_	99‐50‐3	*Cornus officinalis*
Coniferin	67.8	342.1314612	C_16_H_22_O_8_	531‐29‐3	*Cistanche deserticola*
Phillyrin	69.2	534.2101	C_27_H_34_O_11_	487‐41‐2	*Cistanche deserticola*
Cistanoside f	69.9	488.15298	C_21_H_28_O_13_	97,411‐47‐7	*Cistanche deserticola*
Hesperetin	82.8	302.07903	C_16_H_14_O_6_	520‐33‐2	*Cistanche deserticola*
Allantoin	85.4	158.0439886	C_4_H_6_N_4_O_3_	97‐59‐6	*Pueraria lobata*
Scoparone	90.5	206.057906	C_11_H_10_O_4_	120‐08‐1	*Pueraria lobata*
Quercetin	93.5	302.04265	C_15_H_10_O_7_	117‐39‐5	*Cornus officinalis*
Geniposidic acid	101.2	374.12129	C_16_H_22_O_10_	27,741‐01‐1	*Cistanche deserticola*
Swertiamarin	102	374.12129	C_16_H_22_O_10_	17,388‐39‐5	*Cornus officinalis*
Koaburaside	104	332.11073	C_14_H_20_O_9_	41,653‐73‐0	*Dendrobium officinale*
Tyrosol	136.1	138.06808	C_8_H_10_O_2_	501‐94‐0	*Cistanche deserticola*
Salidroside	186.1	300.1209	C_14_H_20_O_7_	10,338‐51‐9	*Cistanche deserticola*
(−)‐pinoresinol	221	358.1416312	C_20_H_22_O_6_	81,446‐29‐9	*Cistanche deserticola*
Secologanin	224.6	388.1369404	C_17_H_24_O_10_	19,351‐63‐4	*Cornus officinalis*
Isoeugenol	235.2	164.0837252	C_10_H_12_O_2_	97‐54‐1	*Cornus officinalis*
Puerarin	240.5	416.11073	C_21_H_20_O_9_	3681‐99‐0	*Pueraria lobata*
Loganin	252.9	390.15259	C_17_H_26_O_10_	18,524‐94‐2	*Cornus officinalis*
Deoxyloganin	254.7	374.15767	C_17_H_26_O_9_	26,660‐57‐1	*Cornus officinalis*
Echinacoside	255.7	786.25823	C_35_H_46_O_20_	82,854‐37‐3	*Cistanche deserticola*
Androsin	261.4	328.11581	C_15_H_20_O_8_	531‐28‐2	*Cistanche deserticola*
Daidzin	269.5	416.11073	C_21_H_20_O_9_	552‐66‐9	*Pueraria lobata*
Verbascoside	271.9	624.20541	C_29_H_36_O_15_	61,276‐17‐3	*Cistanche deserticola*
Epicatechin gallate	273.5	442.0899928	C_22_H_18_O_10_	1257‐08‐5	*Cornus officinalis*
Verbascose	274.9	828.2746692	C_30_H_52_O_26_	546‐62‐3	*Cistanche deserticola*
Caffeic acid	283.5	180.04226	C_9_H_8_O_4_	331‐39‐5	*Cornus officinalis*
Ferulate	286	194.05791	C_10_H_10_O_4_	537‐98‐4	*Dendrobium officinale*
Linalool‐8‐aldehyde	289.5	168.11502	C_10_H_16_O_2_	118,014‐46‐3	*Cornus officinalis*
Formononetin	290.4	268.07356	C_16_H_12_O_4_	485‐72‐3	*Pueraria lobata*
Daidzein	293	254.057906	C_15_H_10_O_4_	486‐66‐8	*Pueraria lobata*
Cornuside	299.3	542.16355	C_24_H_30_O_14_	131,189‐57‐6	*Cornus officinalis*
Syringin	299.3	372.1420254	C_17_H_24_O_9_	118‐34‐3	*Dendrobium officinale*
Menthol	309.6	156.151407	C_10_H_20_O	2216‐51‐5	*Cistanche deserticola*
5‐o‐Methylgenistein	311.6	284.06847	C_16_H_12_O_5_	4569‐98‐6	*Pueraria lobata*
2,6‐di‐*tert*‐Butylphenol	319.2	206.16706	C_14_H_22_O	19,126‐15‐9	*Cistanche deserticola*
Alpha‐curcumene	324.9	202.1721412	C_15_H_22_	4176‐17‐4	*Cornus officinalis*
Ononin	326.2	430.12638	C_22_H_22_O_9_	486‐62‐4	*Pueraria lobata*
6‐Hydroxydaidzein	342.9	270.052821	C_15_H_10_O_5_	17,817‐31‐1	*Pueraria lobata*
2′‐Hydroxygenistein	362.2	286.04774	C_15_H_10_O_6_	1156‐78‐1	*Pueraria lobata*
Phenol	372.6	318.14672	C_18_H_22_O_5_	95,041‐90‐0	*Dendrobium officinale*
Resveratrol	391.3	228.0786402	C_14_H_12_O_3_	501‐36‐0	*Dendrobium officinale*
Castanin	406.1	298.08412	C_17_H_14_O_5_	550‐79‐8	*Cistanche deserticola*
Alpha‐tocopherol	579.1	430.38106	C_29_H_50_O_2_	59‐02‐9	*Cornus officinalis*
Beta‐sitosterol	594.8	414.386145	C_29_H_50_O	83‐46‐5	*Pueraria lobata*
Soyasapogenol b	604.1	458.375975	C_30_H_50_O_3_	595‐15‐3	*Pueraria lobata*
Isoformononetin	645.2	268.0735552	C_16_H_12_O_4_	486‐63‐5	*Pueraria lobata*

### 3.2 Edible Ultra‐Micro Powder Prolonged Alcohol Tolerance Time, Shortened Alcohol‐Induced Sleep Time and Sobriety Time

As indicated in Table 2, the intoxication rate of mice exhibited an increasing trend with the escalation of alcohol intake in each group, leading to a corresponding increase in mortality rate. Notably, at a dosage of 0.14 mL/10 g, the intoxication rate of mice reached 100% while the mortality rate remained at 0%. Consequently, 0.14 mL/10 g was determined as the optimal alcohol dosage for mice.

**TABLE 2:** Acute alcohol tolerance test (*n* = 8).

Alcohol dose (mL/10 g)	Drunkenness rate (%)	Mortality rate (%)
0.10	50	0
0.12	75	0
0.14	100	0
0.16	100	12.5

As demonstrated in Table 3, the alcohol tolerance time was significantly prolonged in the silymarin group, the KingDrink group, and the low, medium, and high dose groups of edible ultra‐micro powder in comparison to the alcohol group (14.30 ± 4.67) min. Furthermore, these treatments also exhibited shorter sleep time and sobriety time when compared to the alcohol group (148.27 ± 2.34) min. These findings indicate that the ultra‐micro powder improves behavioral recovery and attenuates the effects of alcohol intoxication.

**TABLE 3:** Effects of edible ultra‐micro powder on behavioral indicators in mice induced by alcohol (*n* = 8).

Group	Alcohol tolerance time (min)	Sleeping time (min)	Sobriety time (min)
Normal	0.00 ± 0.00	0.00 ± 0.00	0.00 ± 0.00
Alcohol	14.30 ± 4.67^###^	148.27 ± 2.34^###^	162.57 ± 7.12^###^
Silymarin	26.18 ± 5.88*	98.11 ± 4.17**	126.29 ± 9.76**
KingDrink	32.60 ± 2.10**	102.23 ± 5.74**	134.83 ± 7.80**
Low	22.87 ± 6.13*	127.09 ± 4.85*	149.96 ± 9.89*
Medium	27.58 ± 3.22*	118.14 ± 3.04**	146.72 ± 6.50*
High	25.61 ± 7.83*	121.60 ± 1.20**	147.21 ± 9.01*

*Note:* ^###^ *p* < 0.001 versus normal group; **p < 0.01 versus alcohol group; *p < 0.05.

### 3.3 Effects of Edible Ultra‐Micro Powder on Body Weight and Liver Index of Mice Induced by Alcohol

According to the data presented in Table 4, it was observed that the body weight of the alcohol group was significantly lower compared to the Normal group, while the liver index of the alcohol group was significantly higher. Conversely, treatment with medium and high doses of the ultra‐micro powder significantly increased body weight relative to the alcohol group. Furthermore, all powder dosage groups (low, medium, and high) demonstrated a notable reduction in the liver index.

**TABLE 4:** Effects of edible ultra‐micro powder on the change of body weight and liver index in mice induced by alcohol (*n* = 8).

Group	Initial body weight (g)	Final body weight (g)	Liver index (%)
Normal	30.27 ± 1.31	36.20 ± 0.36	4.78 ± 0.22
Alcohol	30.62 ± 1.15	27.30 ± 1.33^##^	5.08 ± 0.67^#^
Silymarin	29.93 ± 1.34	27.37 ± 1.80	4.65 ± 0.14**
KingDrink	29.69 ± 1.52	28.03 ± 0.82	4.73 ± 0.30*
Low	30.91 ± 1.29	28.01 ± 1.06	4.87 ± 0.47*
Medium	29.95 ± 1.23	28.74 ± 1.53*	4.67 ± 0.32**
High	30.27 ± 1.82	28.72 ± 0.94*	4.66 ± 0.84**

*Note:* ^#^ *p* < 0.05 and ^##^ *p* < 0.01 versus Normal group; *p < 0.05, **p < 0.01 versus alcohol group.

### 3.4 Effects of Edible Ultra‐Micro Powder on Serum Hepatic Injury Indexes in Mice

**FIGURE 2:**
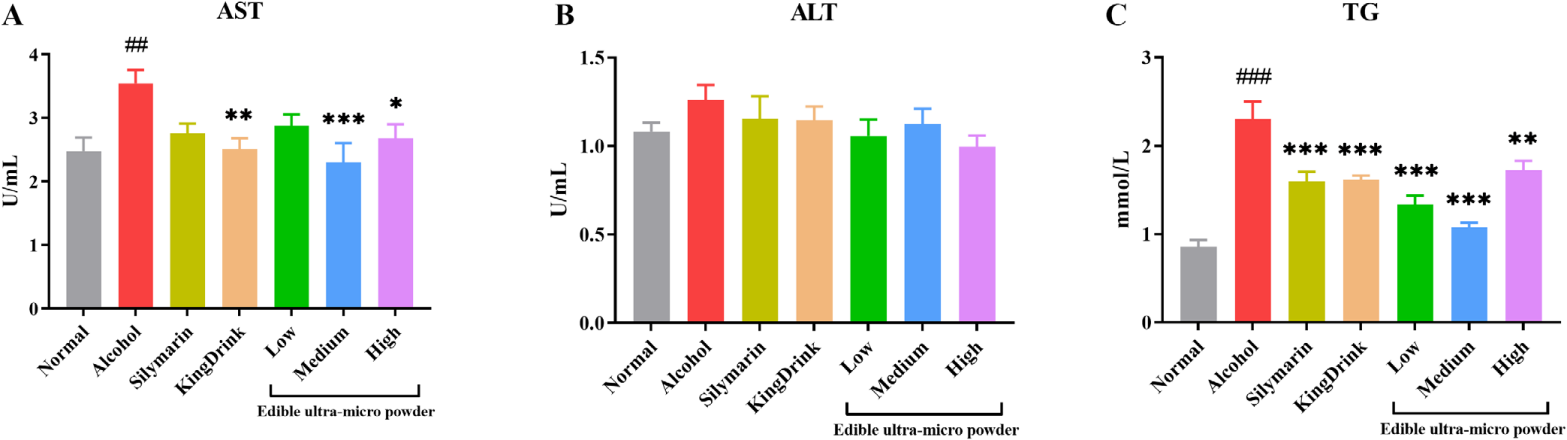
Effects of edible ultra‐micro powder on AST (A), ALT (B), and TG (C) levels in the serum of alcohol‐induced liver injury. ^##^ *p* < 0.01; ^###^ *p* < 0.001 versus normal group. *p < 0.05; **p < 0.01; ***p < 0.001 versus alcohol group. Data are expressed as means ± SEM (*n* = 8).

As shown in Figure 2A, serum AST activity was significantly elevated in the alcohol group compared to the Normal group. However, treatment with silymarin, KingDrink, or medium and high doses of the ultra‐micro powder effectively reduced these AST levels. Although serum ALT levels in the alcohol group were higher than in the Normal group, the difference did not reach statistical significance (Figure 2B). Furthermore, alcohol consumption increased serum TG levels (Figure 2C), which were subsequently reduced following the administration of silymarin or the low‐ and medium‐dose ultra‐micro powder.

### 3.5 Effects of Edible Ultra‐Micro Powder on the Activity of Alcohol Metabolizing Enzymes in Liver

**FIGURE 3:**
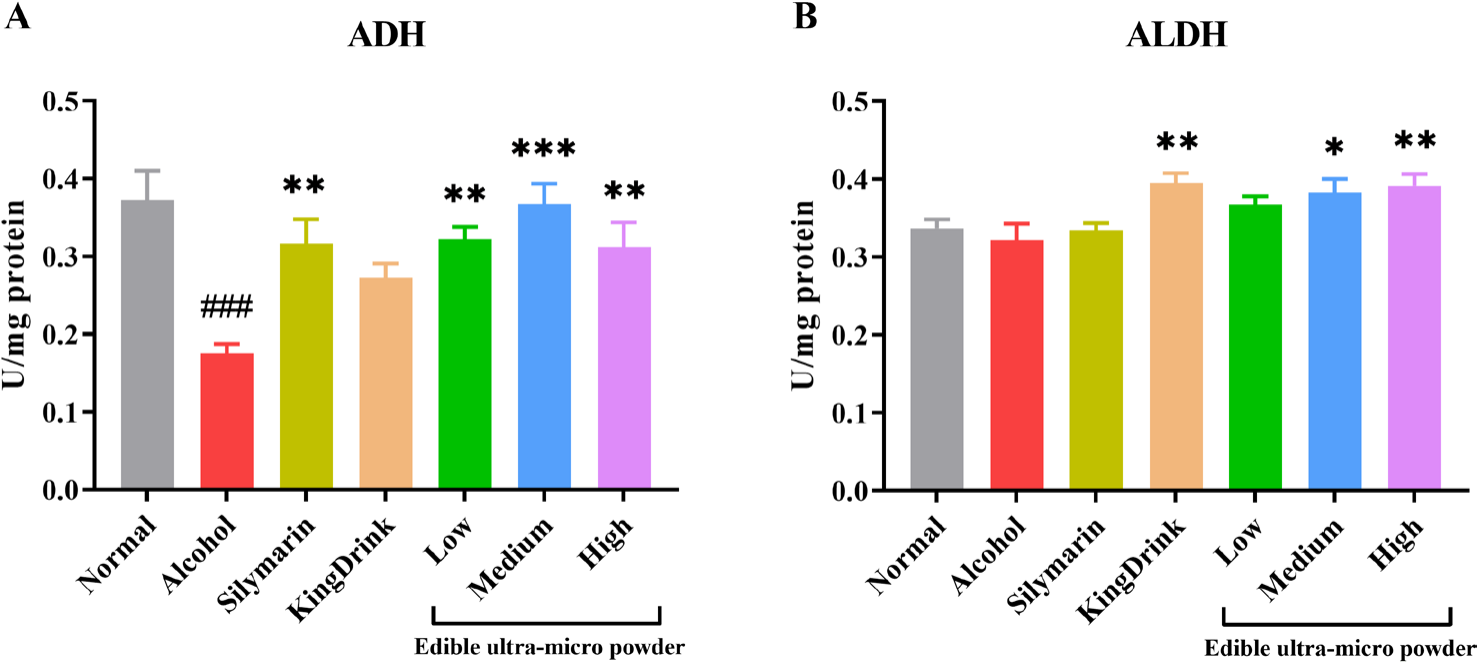
Effects of edible ultra‐micro powder on hepatic ADH (A) and ALDH (B) activity in the liver of alcohol‐induced liver injury. ^###^ *p* < 0.001 versus normal group. *p < 0.05; **p < 0.01; ***p < 0.001 versus alcohol group. Data are expressed as means ± SEM (*n* = 8).

As shown in Figure 3A, hepatic ADH activity in the alcohol group was significantly lower than in the Normal group. However, silymarin and all doses of the ultra‐micro powder significantly increased ADH activity compared to the alcohol group. In contrast, differences in ALDH levels across groups were less pronounced and did not reach statistical significance (Figure 3B).

### 3.6 Effect of Edible Ultra‐Micro Powder on Liver Oxidative Stress Indexes in Mice

**FIGURE 4:**
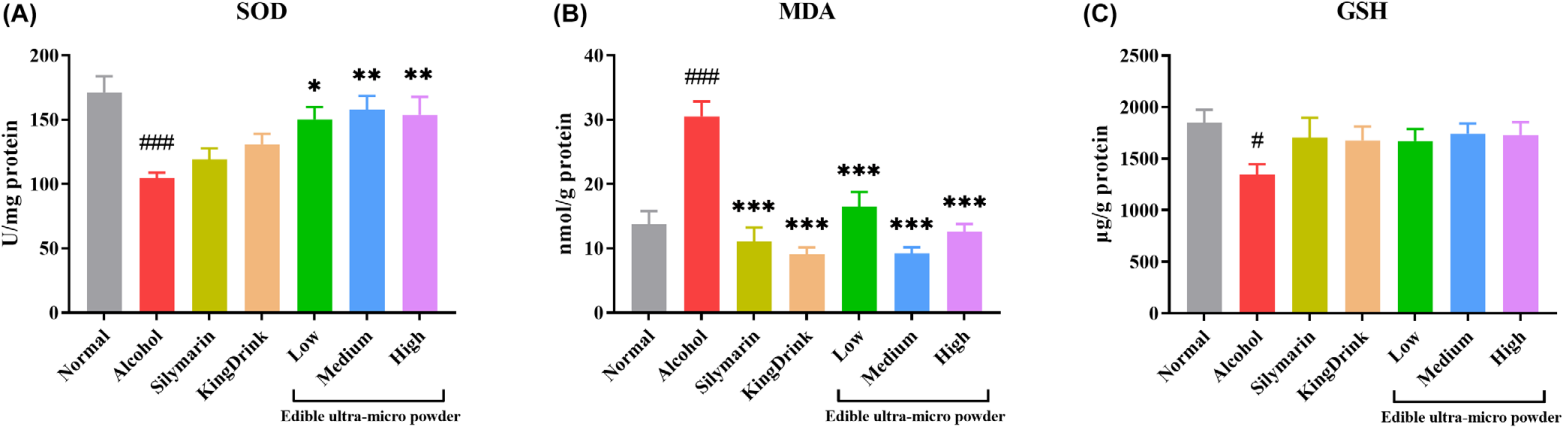
Effects of edible ultra‐micro powder on hepatic SOD (A), MDA (B), and GSH (C) content in the liver of alcohol‐induced liver injury. ^#^ *p* < 0.05; ^###^ *p* < 0.001 versus normal group. *p < 0.05; **p < 0.01; ***p < 0.001 versus alcohol group. Data are expressed as means ± SEM (*n* = 8).

As shown in Figure 4A, hepatic SOD activity in the alcohol group was significantly lower than in the Normal group. However, treatment with the medium dose of ultra‐micro powder significantly increased SOD levels compared to the alcohol group. Figure 4B revealed that MDA levels were significantly elevated in the alcohol group relative to the Normal group. Conversely, both silymarin and the high‐dose ultra‐micro powder significantly reduced MDA content. Figure 4C demonstrated that the GSH content in the alcohol group was lower than in the Normal group, while administration of the ultra‐micro powder did not result in a statistically significant increase in GSH levels.

### 3.7 Effects of Edible Ultra‐Micro Powder on the Contents of Cytokines in Liver

**FIGURE 5:**
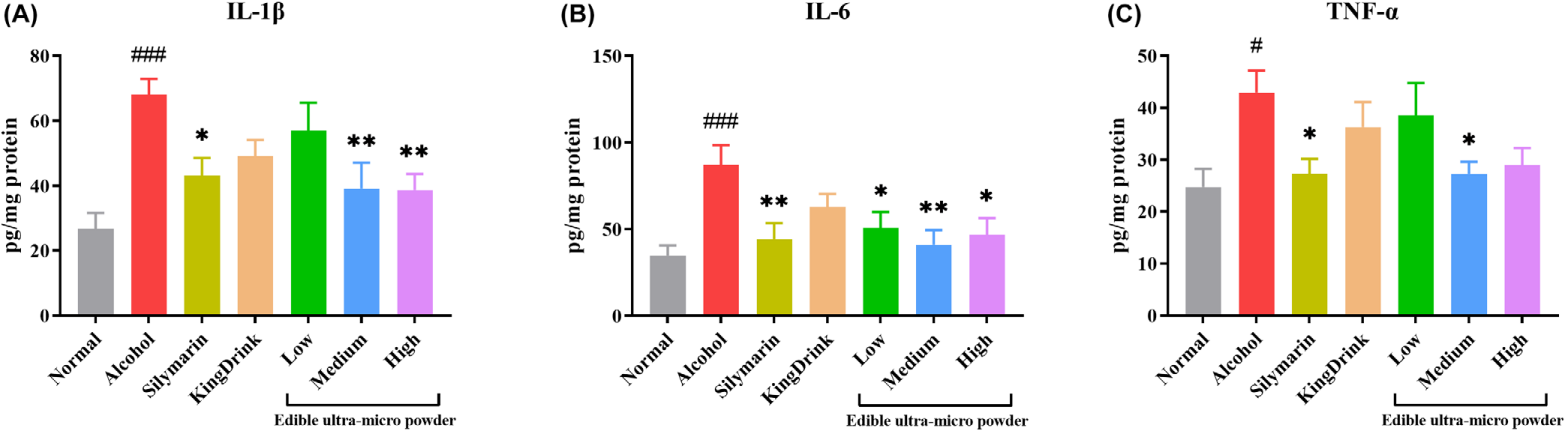
Effects of edible ultra‐micro powder on hepatic IL‐1β (A), IL‐6 (B), and TNF‐α (C) level in the liver of alcohol‐induced liver injury. ^#^ *p* < 0.05; ^###^ *p* < 0.001 versus Normal group. *p < 0.05; **p < 0.01 versus alcohol group. Data are expressed as means ± SEM (*n* = 8).

Proinflammatory cytokines are critical mediators of alcohol‐induced liver injury. Excessive alcohol consumption triggers a hepatic inflammatory response, prompting the release of cytokines such as IL‐1β, IL‐6, and TNF‐α, which drive the progression and severity of tissue damage (Li et al. 2025). As shown in Figure 5, alcohol administration induced the overexpression of these proinflammatory cytokines in the liver. In contrast, treatment with the ultra‐micro powder and control drugs significantly alleviated the release of these inflammatory cytokines.

### 3.8 Effect of Edible Ultra‐Micro Powder on the Histomorphology of Mouse Liver

**FIGURE 6:**
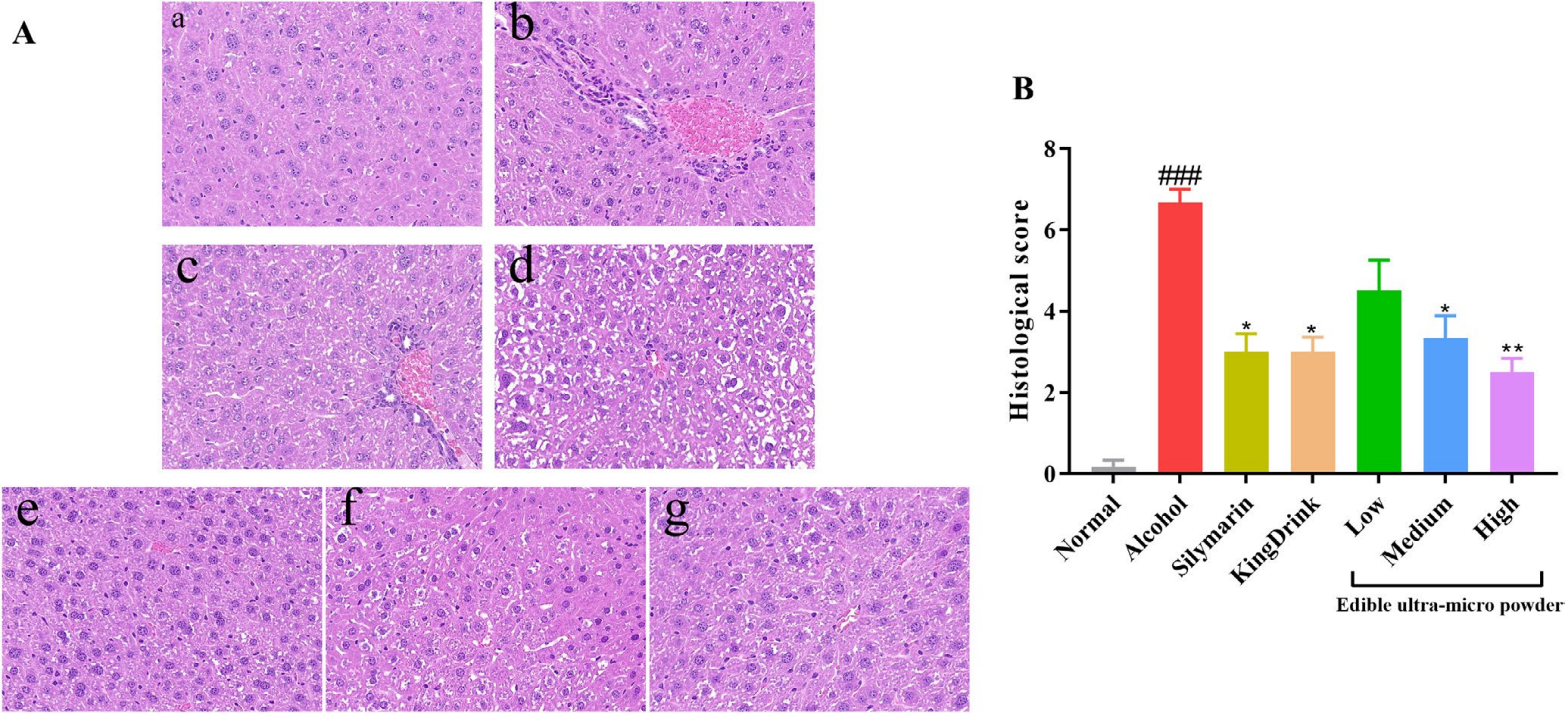
Effects of edible ultra‐micro powder on hepatic histopathology in mice induced by alcohol. (A) Representative liver HE staining images. (a) Normal; (b) Alcohol; (c) Silymarin; (d) KingDrink; (e) Low dose of edible ultra‐micro powder; (f) Medium dose of edible ultra‐micro powder; (g) High dose of edible ultra‐micro powder. (B) Histological score. Data are expressed as means ± SEM (*n* = 6). ^###^ *p* < 0.001 versus normal group. *p < 0.05; **p < 0.01.

To objectively quantify liver injury, a histological scoring system was implemented. Although eight mice were used for initial tissue collection, the quantitative histological score was performed on *n* = 6 representative paraffin sections per group to ensure high‐quality, artifact‐free architectural layout across all non‐overlapping fields. According to Figure 6A, the sections of mouse liver were observed using HE staining, which provides information about blood and tissue structure. The liver tissue of the Normal group (a) exhibited a clear structure, with hepatocytes arranged closely and orderly, and well‐distributed blood vessels and bile ducts. In the alcohol group (b), some hepatocytes displayed poorly defined margins, eosinophilic cell fragments, disrupted lobular structure, and irregular hepatocyte arrangement. In the Silymarin group (c), the liver tissue remained intact, with hepatic cords arranged radially around the central vein. Some hepatocytes exhibited microvesicular steatosis, while the overall structure of the liver lobules appeared normal. Similarly, in the KingDrink group (d), the liver tissue remained intact, with clear cell margins, and some hepatocytes showed microvesicular steatosis. Comparing to the alcohol group, the low‐dose group of edible ultra‐micro powder (e) exhibited microvesicular steatosis in some hepatocytes. Additionally, in the medium‐dose group (f) and high‐dose group (g) of edible ultra‐micro powder, a small number of hepatocytes displayed microvesicular steatosis. However, the liver tissue remained intact with no abnormal cytoplasmic staining. According to Figure 6B, the model group exhibited a high histological score, while Silymarin, KingDrink, and powder at medium and high doses significantly decreased the histological score.

### 3.9 Edible Ultra‐Micro Powder Inhibited TLR4/NF‐κB and NLRP3/ASC Signaling Pathways in the Liver

**FIGURE 7:**
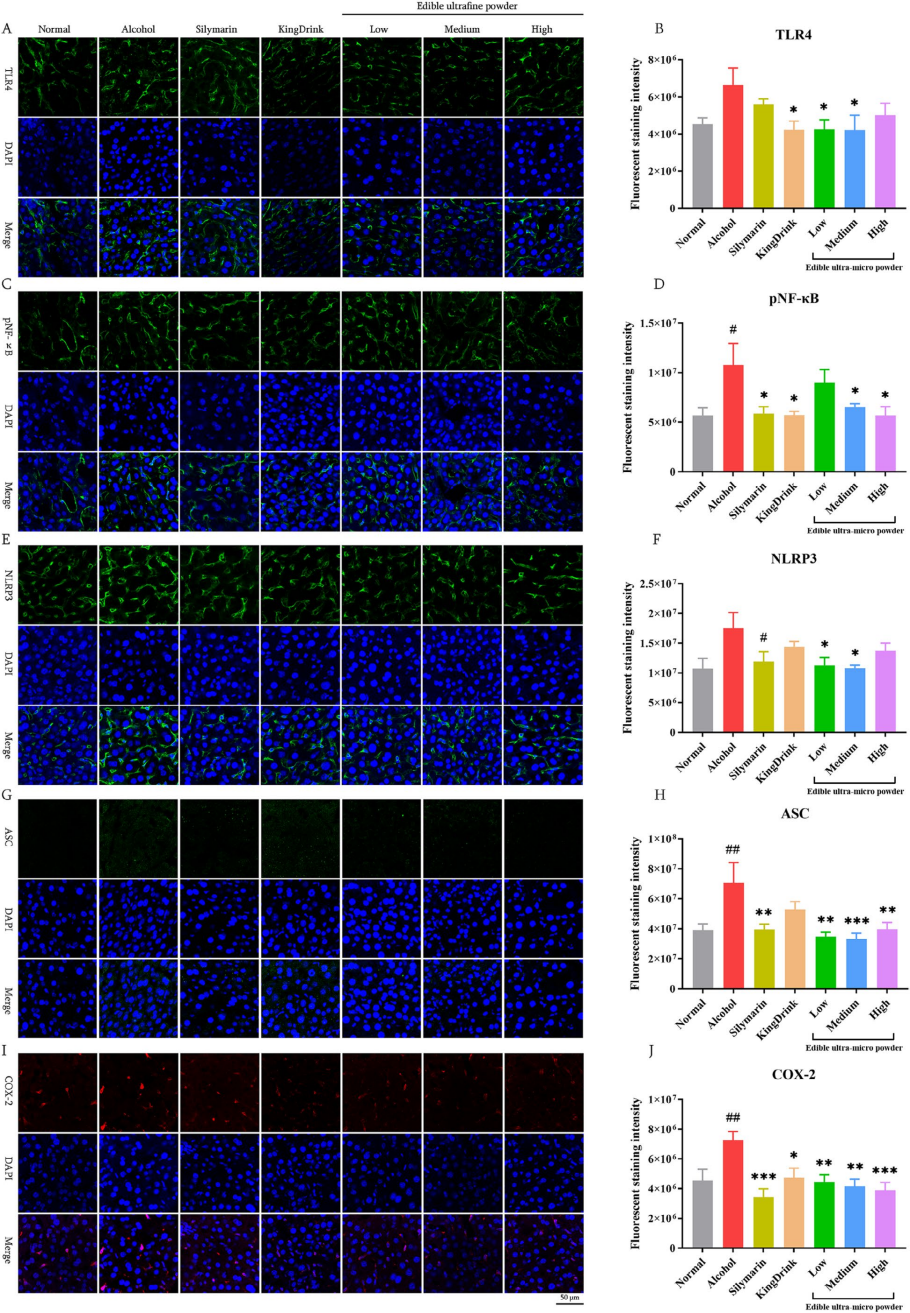
Effects of edible ultra‐micro powder on hepatic TLR4/NF‐κB and NLRP3/ASC signaling pathways in the liver of alcohol‐induced liver injury. (A) Representative image of TLR4 staining. (B) The quantitative analysis of TLR4. (C) Representative image of pNF‐κB staining. (D) The quantitative analysis of pNF‐κB. (E) Representative image of NLRP3 staining. (F) The quantitative analysis of NLRP3. (G) Representative image of ASC staining. (H) The quantitative analysis of ASC. (I) Representative image of COX‐2 staining. (J) The quantitative analysis of COX‐2. ^#^ *p* < 0.05; ^##^ *p* < 0.01 versus Normal group. *p < 0.05; **p < 0.01; ***p < 0.001 versus Alcohol group. Data are expressed as means ± SEM (*n* = 8).

TLR4 is a key immune receptor that activates the NF‐κB pathway during hepatocyte damage (Tang et al. 2025). Our results, as shown in Figure 7A,B, indicate that TLR4 protein expression was significantly higher in the alcohol group compared to the Normal group. However, in the groups treated with the low, medium, and high doses of edible ultra‐micro powder, we observed a notable reduction in TLR4 protein expression. Additionally, Figure 7C,D demonstrates that the pNF‐κB level was significantly elevated in the alcohol group compared to the Normal group, whereas treatment with silymarin and the medium and high doses of the edible ultra‐micro powder effectively decreased pNF‐κB levels.

The NLRP3 inflammasome and its adaptor ASC are critical drivers of the inflammatory response (Ma et al. 2025). According to Figure 7E,F, NLRP3 protein expression was markedly higher in the alcohol group compared to the Normal group. Conversely, all treatments exhibited reduced NLRP3 protein expression in liver tissue compared to the alcohol group. Furthermore, ASC, a major receptor adaptor for the NLRP3 inflammasome, plays a crucial role in its formation and activation. According to Figure 7H,I, the ASC levels were significantly increased in the alcohol group. However, treatment with the edible ultra‐micro powder effectively reduced the ASC protein levels.

COX‐2 is a crucial regulatory enzyme that significantly contributes to the inflammatory response and regulation in vivo (Wang et al. 2022). As shown in Figure 7J,K, the expression of COX‐2 was elevated in the alcohol group compared to the Normal group. However, this increase was significantly attenuated following treatment with the medium dose of ultra‐micro powder.

### 3.10 Edible Ultra‐Micro Powder Reshaped Diversity and Composition of the Intestinal Flora

#### 3.10.1 Phylum Level

**FIGURE 8:**
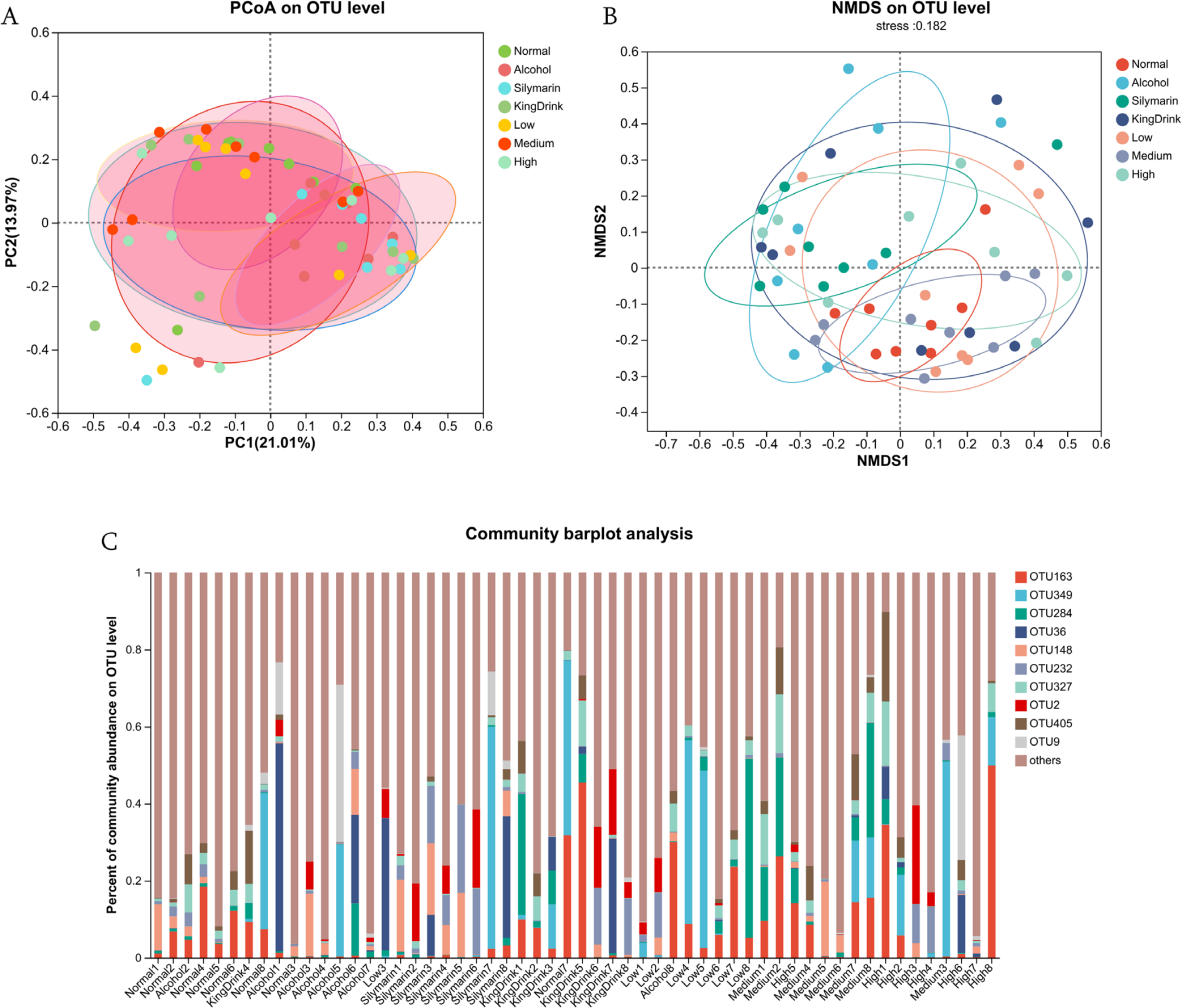
Effects of edible ultra‐micro powder on the intestinal flora at OTU level. (A) β‐Diversity was determined based on PCA. (B) β‐Diversity was determined based on NMDS; (C) The OTU compositions of all samples. Data are expressed as means ± SEM (*n* = 8).

β‐diversity analysis (PCA and NMDS) at the OTU level revealed distinct clustering between the Normal and Alcohol groups (Figure 8C), indicating significant microbial community shifts associated with liver injury. Relative community abundances at the OTU level for all samples are presented in Figure 8C.

At the phylum level, the *Chloroflexi* phylum was uniquely present in the Silymarin group. Comparison of microbial abundances revealed notable shifts across experimental groups. Specifically, alcohol administration significantly increased the abundance of *Proteobacteria* (from 0.28% to 15%) and decreased *Firmicutes* (from 45% to 33%) compared to the Normal group. Conversely, both positive controls and powder treatments reduced *Proteobacteria* levels (Silymarin: 6.2%; KingDrink: 6.7%; Low: 1.0%; Medium: 0.35%; High: 4.3%). Notably, the ultra‐micro powder groups (Low: 63%; Medium: 54%; High: 51%) exhibited *Firmicutes* abundances that more closely resembled the Normal group. These findings suggest that the edible ultra‐micro powder effectively regulates the composition of mouse intestinal flora.

#### 3.10.2 Genus Level

**FIGURE 9:**
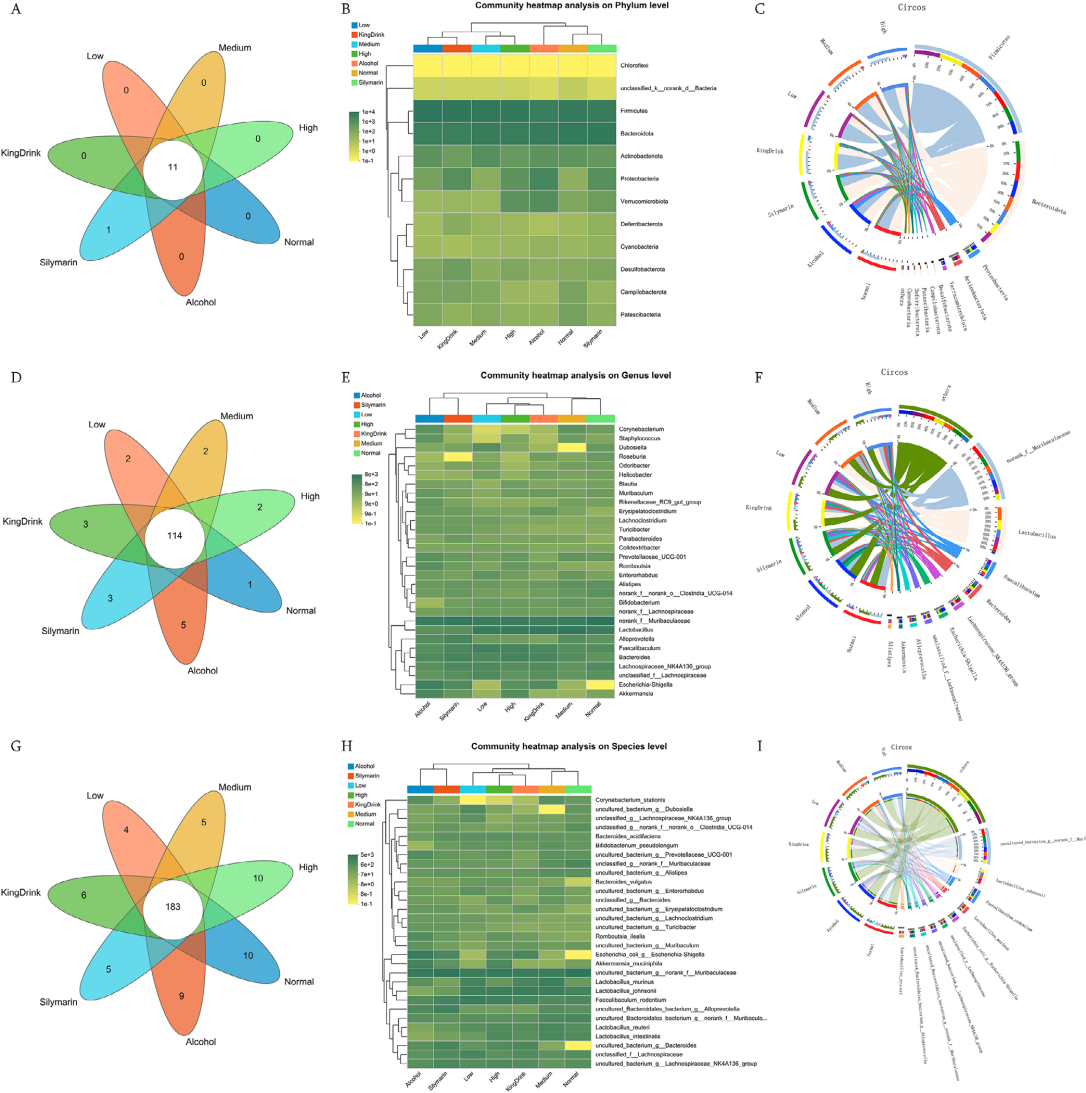
Effects of edible ultra‐micro powder on intestinal flora compositions in liver injury mice. (A) Venn diagram illustrated overlap of phylum in intestinal flora. (B) Community heatmap analysis on phylum level. (C) Proportion of dominant phylum distribution in each group of samples, and proportion of each dominant phylum distribution in different subgroups. (D) Venn diagram illustrated overlap of genus in intestinal flora. (E) Community heatmap analysis on genus level. (F) Proportion of dominant genus distribution in each group of samples, and proportion of each dominant genus distribution in different subgroups. (G) Venn diagram illustrated overlap of species in intestinal flora. (H) Community heatmap analysis on species level. (I) Proportion of dominant phylum distribution in each group of samples, and proportion of each dominant phylum distribution in different subgroups. Data are expressed as means ± SEM (*n* = 8).

As shown in Figure 9D–F, each group displayed a diverse array of microbial species, reflecting the impact of both alcohol and drug treatments on gut composition. Specifically, the abundance of Escherichia‐Shigella surged to 14% in the alcohol group compared to 0.0016% in the Normal group. However, treatment with positive controls or the ultra‐micro powder markedly reduced this abundance (Silymarin: 5.3%; KingDrink: 5.1%; Low: 0.052%; Medium: 0.11%; High: 3.2%).

#### 3.10.3 Species Level

Species‐level analysis (Figure 9G) revealed that alcohol and drug treatments significantly altered the composition of the mouse intestinal flora. As shown in Figure 9H,I, microbial community abundance varied significantly across groups, reflecting the impact of both alcohol consumption and powder administration. In particular, the common probiotics such as *Lactobacillus reuteri* (Normal: 3.1%; Alcohol: 0.39%; Silymarin: 1.3%; KingDrink: 3.3%; Low: 3.0%; Medium: 5.7%; High: 3.6%) and *Lactobacillus johnsonii* (Normal: 7.9%; Alcohol: 0.31%; Silymarin: 0.96%; KingDrink: 12%; Low: 9.5%; Medium: 11%; High: 11%) decreased in Alcohol but increased in KingDrink and edible ultra‐micro powder. On the contrary, the noxious bacteria such as *Escherichia coli* (Normal: 0.0016%; Alcohol: 14%; Silymarin: 5.3%; KingDrink: 5.1%; Low: 0.0052%; Medium: 0.11%; High: 3.2%) increased in Alcohol but decreased in KingDrink and edible ultra‐micro powder.

### 3.11 Difference Analysis in Intestinal Flora at the Phylum, Genus and Species Levels

**FIGURE 10:**
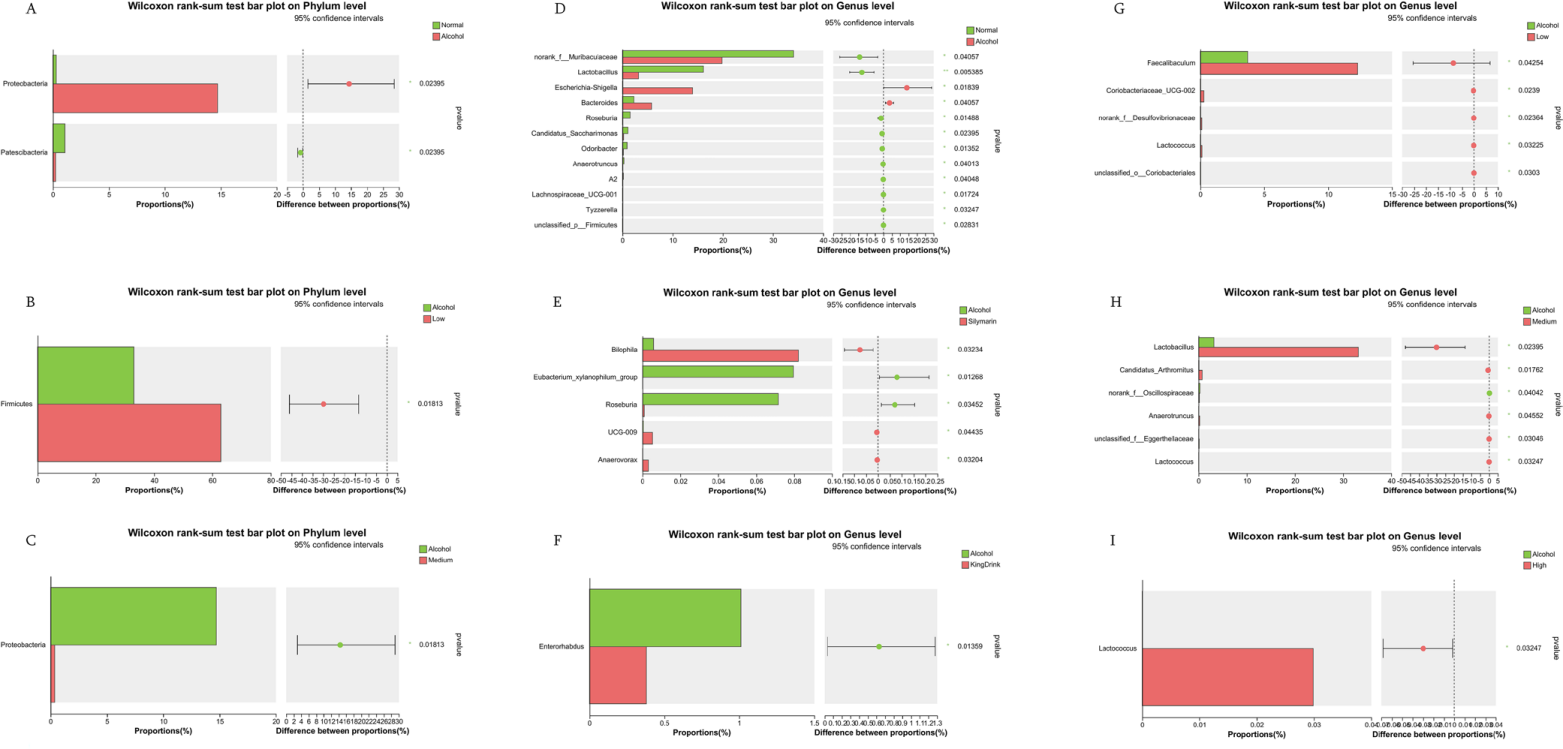
Effects of edible ultra‐micro powder on the significant change of intestinal flora at phylum level and genus level. (A) The difference of phylum level between normal and alcohol. (B) The difference of phylum level between alcohol and low dose of edible ultra‐micro powder. (C) The difference of phylum level between alcohol and medium dose of edible ultra‐micro powder. (D) The difference of genus level between normal and alcohol. (E) The difference of genus level between alcohol and silymarin. (F) The difference of genus level between alcohol and KingDrink. (G) The difference of genus level between alcohol and low dose of edible ultra‐micro powder. (H) The difference of genus level between alcohol and medium dose of edible ultra‐micro powder. (I) The difference of genus level between alcohol and high dose of edible ultra‐micro powder. Data are expressed as means ± SEM (*n* = 8). *p < 0.05; **p < 0.01.

As shown in Figure 10A–C, the relative abundance of Proteobacteria was significantly higher in the alcohol group than in all other groups. Conversely, the low dose of ultra‐micro powder significantly increased Firmicutes abundance, while the medium dose significantly reduced Proteobacteria levels. These results suggest that the medium‐dose intervention modulates Proteobacteria populations, which may contribute to the amelioration of alcohol‐induced liver damage.

Figure 10D–I illustrate significant variability in genus‐level composition across groups. Specifically, the abundances of Muribaculaceae, Lactobacillus, and Roseburia were significantly lower in the alcohol group, while Escherichia‐Shigella and Bacteroides were significantly higher. Treatment effectively reversed these shifts, restoring these genera toward normal levels.

**FIGURE 11:**
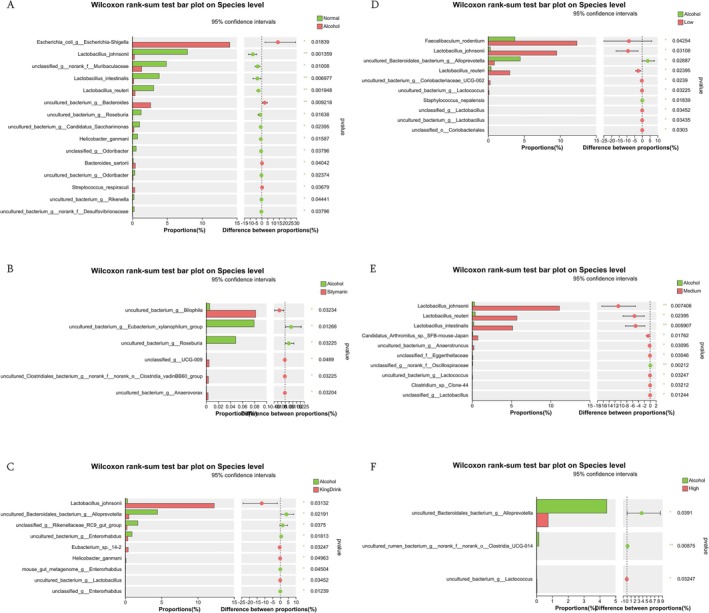
Effects of edible ultra‐micro powder on the significant change of intestinal flora at species level. (A) The difference of species level between normal and alcohol. (B) The difference of species level between alcohol and silymarin. (C) The difference of species level between alcohol and KingDrink. (D) The difference of species level between alcohol and low dose of edible ultra‐micro powder. (E) The difference of species level between alcohol and medium dose of edible ultra‐micro powder. (F) The difference of species level between alcohol and high dose of edible ultra‐micro powder. Data are expressed as means ± SEM (*n* = 8). *p < 0.05; **p < 0.01.

Figure 11 demonstrates significant variability in species‐level composition. Notably, the abundance of Lactobacillus spp. was significantly lower in the alcohol group compared to all other groups. However, ultra‐micro powder intervention typically improved Lactobacillus spp. levels, suggesting that the treatment has a positive impact on restoring these beneficial bacteria within the intestinal flora.

### 3.12 Correlation Analysis Between Intestinal Flora and Serum/Liver Testing Indicators

**FIGURE 12:**
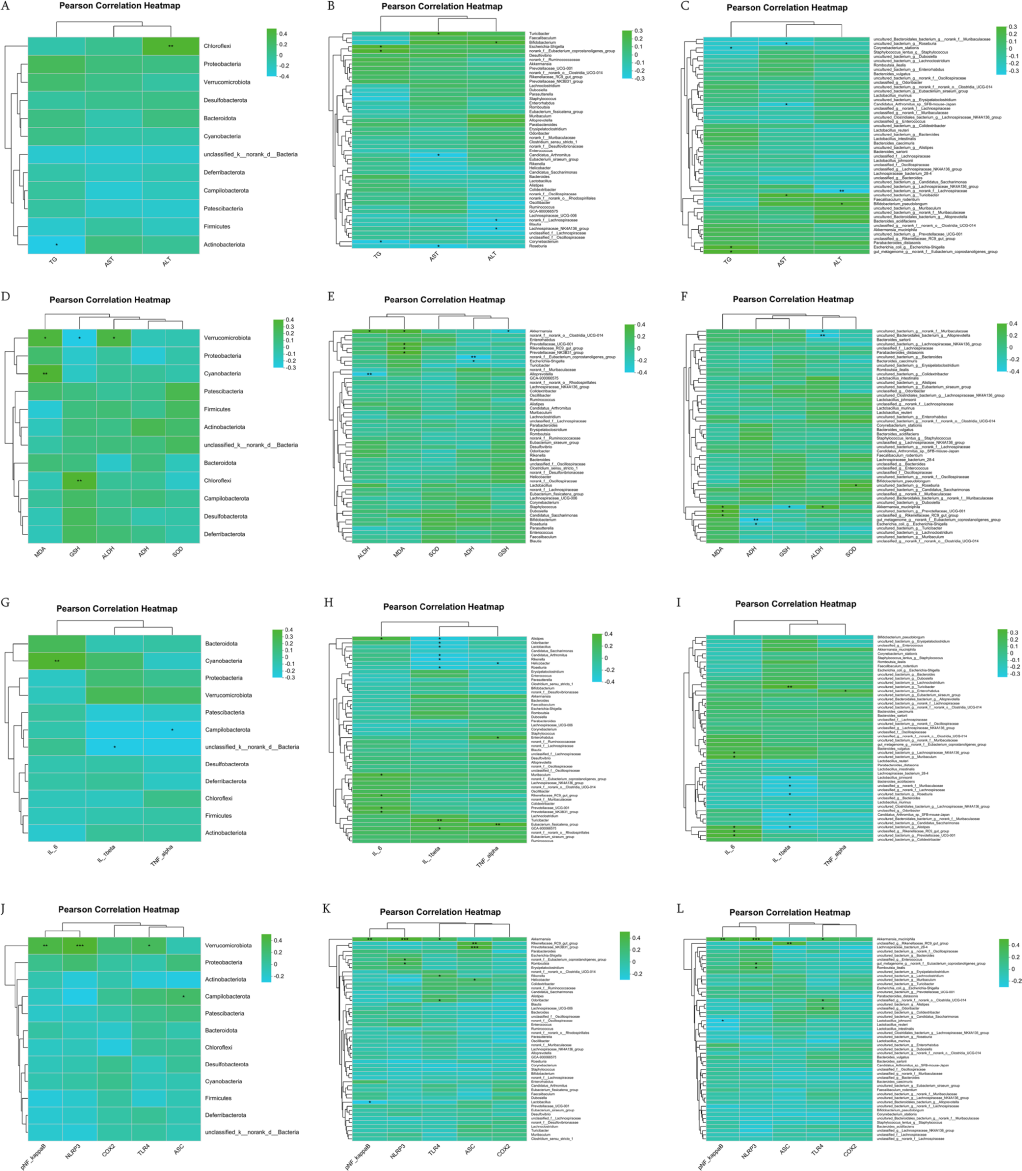
Pearson correlation between relative abundance of intestinal flora and the biochemical indicators. Each cell in the heatmap contains information on the correlation coefficient *r* and *p* value. *r* > 0 indicates positive correlation, expressed in green; *r* < 0 indicates a negative correlation, expressed in blue. (A) Pearson correlation between intestinal flora and AST/ALT/TG at phylum level. (B) Pearson correlation between intestinal flora and AST/ALT/TG at genus level. (C) Pearson correlation between intestinal flora and AST/ALT/TG at species level. (D) Pearson correlation between intestinal flora and MDA/GSH/SOD/ADH/ALDH at phylum level. (E) Pearson correlation between intestinal flora and MDA/GSH/SOD/ADH/ALDH at genus level. (F) Pearson correlation between intestinal flora and MDA/GSH/SOD/ADH/ALDH at species level. (G) Pearson correlation between intestinal flora and IL‐1β/IL‐6/TNF‐α at phylum level. (H) Pearson correlation between intestinal flora and IL‐1β/IL‐6/TNF‐α at genus level. (I) Pearson correlation between intestinal flora and IL‐1β/IL‐6/TNF‐α at species level. (J) Pearson correlation between intestinal flora and TLR4/NLRP3 at phylum level. (K) Pearson correlation between intestinal flora and TLR4/NLRP3 at genus level. (L) Pearson correlation between intestinal flora and TLR4/NLRP3 at species level. *p < 0.05; **p < 0.01. Data are expressed as means ± SEM (*n* = 8).

As shown in Figure 12A–C, the phylum *Chloroflexi* correlated positively with serum ALT, while *Actinobacteriota* correlated negatively with serum TG. At the genus level, *Escherichia‐Shigella* and *Eubacterium_coprostanoligene* showed positive correlations with serum TG, whereas *Corynebacterium* was negatively correlated. Furthermore, *Turicibacter* was positively and *Candidatus_Arthromitus* was negatively correlated with serum AST. *Bifidobacterium* and *Lachnospiraceae* exhibited positive and negative correlations with serum ALT, respectively.

As shown in Figure 12D–F, the phyla *Verrucomicrobiota* and *Cyanobacteria* correlated positively with MDA. *Chloroflexi* correlated positively with GSH, while *Verrucomicrobiota* correlated negatively with GSH but positively with ALDH. At the genus level, *Akkermansia* correlated positively and *Alloprevotella* negatively with ALDH. *Akkermansia*, *Prevotellaceae*, and *Rikenellaceae* were positively linked to MDA. Additionally, *Eubacterium_coprostanoligenes* and *Escherichia‐Shigella* correlated negatively with ADH, and *Akkermansia* correlated negatively with GSH.

For liver inflammatory indicators, as depicted in Figure 12G–I, the phylum Cyanobacteria was positively correlated with IL‐6. The phylum Bacteria was negatively correlated with IL‐1β, and the phylum Campilobacterota was negatively correlated with TNF‐α. The genus Alistipes, Muribaculum, Rikenellacea and Prevotellaceae were positively with IL‐6. The genus Turicibacter and GCA‐900066575 were positively correlated, while Alistipes, Odoribacter, Lactobacillus, Candidatus_Arthromitus, Rikenella, and Roseburia were negatively correlated with IL‐1β. Enterorhabdus and Eubacterium_fissicatena were positively correlated, while Helicobacter was negatively correlated with TNF‐α.

For liver inflammatory signaling pathway, as depicted in Figure 12J–L, the phylum Verrucomicrobiota was positively correlated with TLR4, pNF‐κB, and NLRP3. The phylum Campilobacterota was positively correlated with ASC. The genus Akkermansia, Rikenella, and Odoribacter were positively correlated with TLR4. The genus Akkermansia was positively correlated with pNF‐κB. The genus Akkermansia, Eubacterium_coprostanoligenes, and Romboutsia were positively correlated with NLRP3. Rikenellaceae, Prevotellaceae, and Helicobacter were positively correlated with ASC.

Collectively, these findings suggest that members of the *Verrucomicrobiota* and *Lactobacillus* are closely associated with the hepatoprotective improvements observed following ultra‐micro powder administration.

## 4 Discussion

Alcoholic liver injury remains a major global public health challenge, underscoring the need for effective therapeutic interventions. This study evaluated the potential of an edible ultra‐micro powder composed of *Pueraria lobata* , *Cornus officinalis* , *Cistanche deserticola* , and *Dendrobium officinale* to attenuate hepatic damage and enhance alcohol tolerance. Our findings provide critical observations into the protective mechanisms of this formulation against alcohol‐induced liver injury.

A primary outcome of this study was the significant improvement in alcohol tolerance following the administration of the ultra‐micro powder. The prolonged duration of tolerance suggests an enhanced capacity to metabolize ethanol and withstand its systemic toxicity. This is further supported by the reduction in sleep and sobriety times, indicating that the formulation accelerates behavioral recovery and mitigates alcohol‐induced sedation. By shortening the duration of sedation, this powder may facilitate faster functional recovery and diminish the acute impairments associated with excessive alcohol consumption.

Excessive alcohol intake drives liver damage characterized by elevated serum enzymes and lipid abnormalities (Azahar et al. 2025). Our results revealed a significant decrease in serum AST and TG levels in the groups treated with the edible ultra‐micro powder. These findings indicate a protective effect of the edible ultra‐micro powder against alcohol‐induced liver damage. The reduction in serum AST levels suggests a preservation of liver function, as AST is an enzyme primarily located in hepatocytes and released into the bloodstream upon liver injury. Moreover, the decrease in TG levels suggests an amelioration of alcohol‐induced lipid abnormalities, which are closely linked to liver steatosis and inflammation (Jeon and Carr 2020). Beyond biochemical markers, we assessed the hepatic oxidative stress status. Alcohol consumption generates ROS, leading to tissue damage (Tsermpini et al. 2022). However, the ultra‐micro powder significantly decreased MDA levels, a key indicator of lipid peroxidation, while increasing SOD activity. These results suggest that the powder enhances the liver's antioxidant defense mechanisms, effectively mitigating oxidative damage (H. Gan et al. 2025). Furthermore, our study investigated the activity of ADH and ALDH, a key enzyme involved in alcohol metabolism. We found that the edible ultra‐micro powder increased the activity of ADH and ALDH in liver tissues. This observation suggests that the edible ultra‐micro powder may enhance alcohol metabolism, leading to a more efficient breakdown and clearance of alcohol from the body. By promoting alcohol metabolism, the ultra‐micro powder may alleviate the toxic effects of alcohol on the liver. On the other hand, when the liver is exposed to various insults such as viral infections, alcohol abuse, drugs, toxins, or autoimmune processes, it triggers an inflammatory response (Kostallari et al. 2025). During this response, proinflammatory cytokines are released and contribute to the development and progression of liver injury. These cytokines can directly damage liver cells and disrupt normal liver function (Yu et al. 2026). They contribute to hepatocyte apoptosis, necrosis, and oxidative stress, leading to tissue injury (Shen et al. 2025). In this study, the upregulation of these cytokines in the alcohol group was significantly reversed by the ultra‐micro powder, indicating that its hepatoprotective effects are closely tied to the suppression of alcohol‐induced inflammatory responses.

To investigate the mechanisms underlying the protective effects of the ultra‐micro powder, we examined key protein expression associated with the hepatic inflammatory response. The TLR4/NF‐κB and NLRP3/ASC pathways are critical drivers of alcohol‐induced liver damage, often forming a self‐amplifying inflammatory cycle that recruits immune cells and exacerbates tissue injury (Chen et al. 2026; Gong et al. 2026). Consequently, targeting these signaling axes has been proposed as a primary therapeutic strategy for managing hepatic disorders (Wen et al. 2021). For example, ihibiting TLR4 or blocking NLRP3 inflammasome activation are potential approaches to modulate the inflammatory response and attenuate liver damage (Song et al. 2026; Wang, Bian, et al. 2025). Our findings revealed that the ultra‐micro powder significantly reduced the expression of TLR4, pNF‐κB, NLRP3, and ASC. By modulating these key proteins, the formulation effectively attenuates the inflammatory cascade triggered by alcohol consumption. These results provide significant insight into the molecular mechanisms of the ultra‐micro powder and support its potential as a therapeutic intervention for mitigating alcoholic liver injury.

The gut microbiota is a critical regulator of the immune system. Dysbiosis can trigger abnormal intestinal immune responses (Zheng et al. 2020), releasing cytokines and chemokines into the portal circulation that directly contribute to liver injury (Robinson et al. 2016). Furthermore, gut dysbiosis promotes the recruitment of neutrophils and macrophages to the liver, exacerbating inflammation and tissue damage (Zhou et al. 2026). Understanding these interactions is essential for developing therapeutic strategies that target the gut‐liver axis. In this study, the edible ultra‐micro powder primarily modulated the abundance of Proteobacteria at the phylum level, and Lactobacillus and Escherichia‐Shigella at the genus level. Certain *Proteobacteria*, such as *Helicobacter*, have been linked to chronic liver disease and the progression of fibrosis (Okushin et al. 2018; Waluga et al. 2015). Conversely, Lactobacillus species exert hepatoprotective effects by strengthening the gut barrier, modulating the immune system, and producing antimicrobial substances (Y. Gan et al. 2021; Jeong et al. 2022). Finally, pathogenic strains of Escherichia‐Shigella can invade the intestinal epithelium and disseminate systemically, inducing a host immune response that drives hepatic damage (Nie et al. 2021; Wu et al. 2022). By restoring the balance between these beneficial and pathogenic taxa, the ultra‐micro powder provides a systemic approach to mitigating alcohol‐induced liver injury.

Finally, we analyzed the correlation between the intestinal microbiota and hepatic functional and inflammatory indicators. Our results revealed that the phylum Verrucomicrobiota and the genus Akkermansia were widely correlated with improvements in liver health. Members of the genus Akkermansia are specialized mucin‐degraders that play a vital role in maintaining the integrity and thickness of the gut mucus layer (Hansson 2019; Raimondi et al. 2021). By reinforcing the gut barrier, *Akkermansia* prevents the translocation of pathogens and toxins into the systemic circulation, thereby alleviating hepatic inflammation (Shaheen et al. 2025). Additionally, Verrucomicrobiota can modulate pro‐inflammatory signaling pathways to promote immune homeostasis (Lindenberg et al. 2019). In addition, the famous genus *Lactobacillus* was also shown to be correlated with different indicators. *Lactobacillus* species play a crucial role in maintaining a healthy gut microbiota. They help create an environment that supports the growth of beneficial bacteria while inhibiting the growth of harmful microorganisms (J. Yang et al. 2026). *Lactobacillus* species produce lactic acid, which helps lower the pH of the gut, making it less favorable for the growth of pathogens. This promotes a balanced microbial community and supports overall gut health. *Lactobacillus* can also interact with the immune system and modulate immune responses (Jeong et al. 2022). They can stimulate the production of anti‐inflammatory cytokines while suppressing pro‐inflammatory cytokines. This immunomodulatory effect helps maintain immune homeostasis, reduces inflammation, and supports immune function. These observations clearly indicated Verrucomicrobiota and *Lactobacillus* were closely associated with the restoration of liver injury by this edible ultra‐micro powder.

Although our findings provide compelling evidence that the ultra‐micro powder modulates the gut‐liver axis, this study has notable limitations. First, we did not utilize germ‐free or antibiotic‐treated mice to definitively establish whether the observed hepatoprotection is entirely microbiota‐dependent. Future research employing fecal microbiota transplantation (FMT) or germ‐free models is warranted to elucidate the direct causal mechanisms by which these edible plants regulate systemic metabolism and inflammation. Consequently, the hypothesis that this powder acts as a multi‐organ functional food remains preliminary. Second, while we focused on physiological outcomes of oxidative stress—such as MDA reduction and SOD modulation—the upstream molecular signaling requires further investigation. Nuclear factor erythroid 2‐related factor 2 (Nrf2) is the master regulator of the antioxidant response, and it is plausible that the ultra‐micro powder exerts its effects via the Nrf2 pathway. Future studies will focus on quantifying Nrf2 expression and nuclear translocation to further clarify the comprehensive molecular mechanisms of this formulation.

## 5 Conclusions

In conclusion, our study demonstrates that the edible ultra‐micro powder comprising *Pueraria lobata, Cornus officinalis, Cistanche deserticola*, and *Dendrobium officinale* possesses significant therapeutic potential against alcoholic liver injury. The consumption of this edible ultra‐micro powder resulted in improved alcohol tolerance, enhanced antioxidant defense, and decreased inflammatory response. More importantly, the results demonstrated the crucial role of phylum Verrucomicrobiota and genus *Lactobacillus* for the nutritional regulation, which are linked to in the modulation of gut‐liver axis. These findings indicate the potential of this plant‐based intervention as a natural and effective approach to attenuate the detrimental effects of excessive alcohol consumption. Further research, including clinical trials, is warranted to validate these findings and explore the detailed mechanisms underlying the protective effects of this edible ultra‐micro powder.

## Author Contributions

**Jia‐Yu Huang:** investigation, formal analysis, writing – original draft. **Xin‐Yu Wang:** investigation, formal analysis. **Li‐Tao Yi:** conceptualization, funding acquisition, supervision, writing – review and editing. **Jie Cheng:** conceptualization, investigation. **Guang‐Hui Xu:** conceptualization, writing – review and editing. **Wei‐Feng Huang:** conceptualization, investigation, writing – review and editing, writing – original draft.

## Funding

This work was supported by Science and Technology Foundation of Nanping city (N2024K007).

## Ethics Statement

All experimental procedures received prior approval from the Huaqiao University Institutional Animal Care and Use Committee, Approval No. A2023027, and were conducted in strict accordance with guidelines set by the China Council on Animal Care.

## Conflicts of Interest

The authors declare no conflicts of interest.

## Supporting information


**File S1:** The identified information of constituents from the edible ultra‐micro powder.

## Data Availability

The data that support the findings of this study are available from the corresponding author upon reasonable request.
